# Impact of gaps in care for malnourished patients on length of stay and hospital readmission

**DOI:** 10.1186/s12913-019-3918-3

**Published:** 2019-02-01

**Authors:** Joanna Bryan Ringel, Deanna Jannat-Khah, Rachel Chambers, Emily Russo, Louise Merriman, Renuka Gupta

**Affiliations:** 1 Food and Nutrition, New York-Presbyterian Hospital, 525 East 68th street, New York, NY 10065 USA; 2 Division of General Internal Medicine, Weill Cornell Medical College, 525 East 68th street, Box 331, New York, NY 10065 USA

**Keywords:** Malnutrition, Discharge, Communication, Length of stay, Readmission

## Abstract

**Background:**

Few published articles have focused on identifying the gaps in care that follow a malnutrition diagnosis and their effects on length of stay (LOS) and 90-day readmission. We hypothesized that length of stay and readmission were associated with these gaps in care.

**Methods:**

Two registered dietitians retrospectively reviewed charts of 229 adult malnourished patients admitted to a medicine unit to determine their system level gap in care: communication, test delay, or discharge planning. In this secondary analysis, both readmission and length of stay were regressed on each gap in care.

**Results:**

Any system level gap was associated with a greater length of stay (β: 1.48, 95% CI: 1.15–1.91) and specifically the gap related to procedure/testing (β: 2.01, 95% CI: 1.62–2.47) resulted in a two-fold increase in length of stay. There was no association between 90-day readmission and any of the gaps in care.

**Conclusions:**

There was a strong association between those who had any gap in their care and increased length of stay. Mitigating gaps in care may decrease length of stay and, in turn, result in less risk of infection and could potentially lead to reduced healthcare costs.

## Background

Malnourished patients are a population with documented longer length of stay, higher costs, and in-hospital complications [1]. Malnutrition is common among hospital patients with prevalence estimates between 20 and 50% [2]. The extant literature provides evidence that malnutrition is associated with increased length in stay and hospital readmission [1, 3–5]. A recent study in a tertiary hospital analyzed the relationship between nutritional status of 818 adults and hospitalization outcomes. They found that patients who were malnourished had higher 15-day readmission and length of stay (6.9 vs. 4.6). [4] Research shows that these length in stay and readmission disparities can be improved with identification and care of the malnourished. [3] However, research investigating the quality of care that is given to malnourished patients and its impact on length of stay and hospital readmission is lacking. In our previous study, we identified a cohort of malnourished patients and retrospectively assessed their nutrition management. Suboptimal malnutrition management, referred to as gaps in care, were defined as inconsistent discharge instructions, dietitian-doctor miscommunication and excessive length of time spent fasting (nil per os) before procedures/testing while in the hospital. Suboptimal malnutrition management was common, with 76% of these patients having at least one gap in their care. Using the same sample, in this secondary analysis, we hypothesized that these gaps were associated with increased length of stay and increase in 90-day readmission [6].

## Methods

Data were collected retrospectively by two registered dietitians from the medical charts of 229 malnourished patients. Eligibility criteria included age > 18 years, admission to a medicine unit at NewYork Presbyterian-Weill Cornell Medical Center between September 1–November 30, 2014 and diagnosis of malnutrition (ICD9 code 262 or 263.0). Patients who were admitted to the intensive care unit at any point during their hospital stay were excluded. Additionally, 13 patients who died during hospital stay were excluded from the analysis.

Both registered dietitians reviewed the first 43 charts. Overall and Individual Cohen’s Kappa was calculated for all questions to assess interrater reliability. Questions with lower agreement were identified and discussed to reduce future inconsistencies.

Gaps in care were categorized as communication, testing/procedure, and discharge related. A gap was defined as communicative in nature if approval or implementation of an appropriate nutritional recommendation by a dietitian was delayed. Procedure/testing gaps were identified as instances where the malnourished patient was prevented from receiving nutrition due to prolonged *nil* per os (NPO) orders. Inconsistencies between discharge diet instructions and dietitian’s recommendations were considered discharge gaps. The two main outcomes of interest were length of stay and readmission. Length of stay was defined as the number of days between admission and discharge. Readmission was defined as those malnourished patients who were readmitted within 90 days of initial admit date. 90-day readmission was selected due to an insufficient number of patients with 30-day readmission during our data collection period. Additionally, 90-day readmission aligns with the CMS bundled payment plan. This plan links payments of all services a patient receives for a clinical episode and 90 days after discharge. [7] In addition to the main exposures and outcomes described above, data were collected on age, gender, previous medical history, malnutrition severity, and malnutrition context—defined by American Society for Parenteral and Enteral Nutrition/The Academy of Nutrition and Dietetics. [8]

Distributions of continuous variables were investigated and median, inter-quartile range was reported alternatively for variables that were skewed. Logistic regression was used to assess an association between each group in care and 90-day readmission. Due to its skewed distribution, length of stay was log transformed and then linearly regressed on gaps of care. Both crude and full models were developed, adjusting for age, gender, malnutrition severity and malnutrition context. A 95% confidence interval was computed for all adjusted odds ratios and Exponentiated coefficients. *P*-values with an alpha less than 0.05 were considered significant. Stata/MP 14.1 was used for all statistical analyses. This study was approved by the Weill Cornell Medical College Institutional Review Board.

## Results

Pooled Cohen’s Kappa (0.811) of the first 43 chart reviews indicated almost perfect agreement. Among the 229 adult malnourished patients admitted to medicine, the median age of patients was 67 (IQR 54.0, 81.0) years old. Half of the sample was female (50.7%). A majority of the patients had severe malnutrition (80.8%) and were identified as malnourished in the context of chronic disease (79.0%). The median length of stay for all participants was 7 days (IQR 4.0, 14.0) and 82 participants (33.9%) were readmitted within 90 days (Table 1).

**Table 1:** Characteristics of 229 malnourished patients admitted to a medicine unit

Characteristic	N (%)
N	229
Gender	
Female	116 (50.7%)
Male	113 (49.3%)
Age (years) median (IQR)	67.0 (54.0, 81.0)
Body Mass Index median (IQR)	20.1 (17.8, 23.3)
Previous medical history	
Gastrointestinal	129 (56.3%)
Oncology	97 (42.4%)
Respiratory	45 (19.7%)
Endocrine	77 (33.6%)
Cardiac	122 (53.3%)
Orthopedic	46 (20.1%)
Altered Mental Status/Psychiatric/Behavioral, Social	42 (18.3%)
Geriatrics	15 (6.6%)
Malnutrition	4 (1.7%)
Stroke/ Neurology	21 (9.2%)
Pressure Ulcers/Wound Care	3 (1.3%)
Failure to Thrive	2 (0.9%)
Other	119 (52.0%)
Readmitted within 90 days	
Yes	82 (35.8%)
No	147 (64.2%)
Malnourishment severity	
Severe	185 (80.8%)
non-severe	44 (19.2%)
Malnutrition context	
Acute	37 (16.2%)
Chronic	181 (79.0%)
Behavioral	11 (4.8%)
Length of hospital stay median (IQR)	7.0 (4.0, 13.0)

Table 2 displays results of the logistic regression models for readmission and linear regression results of length of stay. Having any gap in care was significantly associated with a 48% increase in length of stay after adjustment (β: 1.48, *p*-value < 0.01). Specifically, patients with a communication gap were associated with a 60% greater length of stay controlling for all the covariates above (β: 1.60, *p*-value < 0.01). Malnourished patients with a testing/procedure gap were significantly associated with a two-fold increase in length of stay in the fully adjusted model (β: 2.01, *p*-value < 0.01). Discharge gaps were not significantly associated with length of stay, (β: 1.06, *p*-value = 0.64). Although the odds of 90-day readmission were higher for those with any gap compared to those without a gap, the difference was insignificant (Table 2).

**Table 2:** Effect of gaps in care on readmission and length of stay

Type of Gap in Care	Any gap	Discharge	Testing/Procedures	Communication
n Gap/Total	184/229	165/229	63/229	31/229
Readmission OR^a^				
Crude	1.29 (.64–2.61)	1.46 (.79–2.72)	1.26 (.69–2.28)	.98 (.45–2.17)
Model 1	1.35 (.66–2.75)	1.61 (.85–3.05)	1.17 (.64–2.15)	1.11 (.49–2.51)
Full Adjusted	1.29 (.61–2.67)	1.50 (.78–2.87)	1.23 (.66–2.30)	1.06 (.46–2.46)
Length of Stay β^a^				
Crude	1.51 (1.17–1.94)***	1.07 (.85–1.34)	1.98 (1.61–9.32)***	1.64 (1.22–2.44)***
Model 1	1.51 (1.17–1.94)***	1.06 (.84–1.34)	2.02 (1.63–2.50)***	1.65 (1.22–2.23)***
Full Adjusted	1.48 (1.15–1.91)***	1.06 (.83–1.32)	2.01 (1.62–2.47)***	1.60 (1.19–2.16)***

Model 1 adjusted for age and gender

Exponentiated coefficients presented for log transformed length of stay

Full-Adjusted model adjusted for model 1 characteristics, malnutrition context, and malnutrition severity

^a^Estimates provided with 95% CI

***indicates p-value < 0.01

**Fig. 1:**
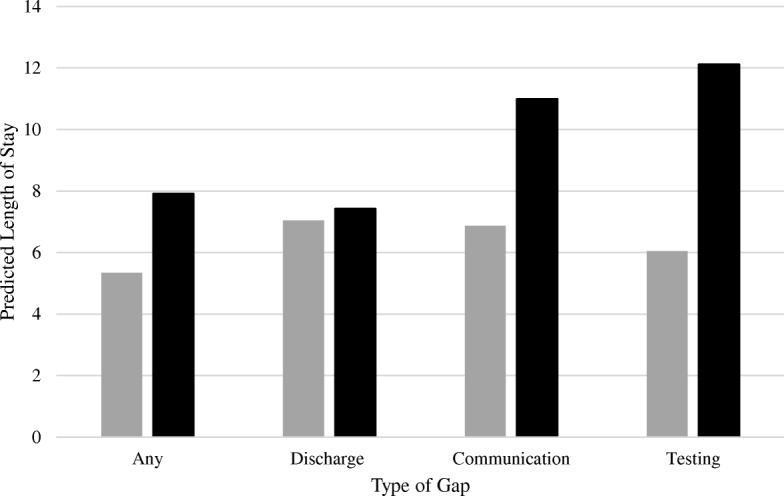
Adjusted predicted length of stay for patients with (black) and without (gray) a gap in care, using mean values of age, gender, malnutrition context and severity

Figure 1 shows the predicted length of stay for those with and without a gap in care. The average participant with any gap in care had 2.5 days longer length of stay compared to someone without any gap in care. Most strikingly, those with a testing/procedure related gap in care had 6 days longer length of stay.

## Discussion

We aimed to assess the impact of gaps of care in length of stay and readmission. There was a strong association between those who had any gap in their care and increased length of stay. The association remained in the fully adjusted model indicating that this relationship exists independent of age, gender, malnutrition context and malnutrition severity. This connection is striking considering that 184 of the 229 participants in our sample had at least one miscommunication, testing/procedure delay or discharge issue during their stay.

There is support for our results in the literature. A retrospective chart review of 37 patients noted that adherence to the advice of Registered Dietitians was associated with reduced length of stay (Mean LOS of 15.78 vs 23.14). [9] A study at an acute-care facility in northwest Louisiana had similar findings. [10] A multidisciplinary team approach intervention on 400 malnourished patients at John Hopkins lead to a reduction in implementation delays and decreased LOS of 1.9 days. [11] In a subset with severe malnutrition, the intervention decreased length of stay by 3.2 days. [11] Some research has linked inappropriately prolonged preoperative fasting to poor health outcomes. An analysis of *nil* per os patients admitted at the Complejo Asistencial Univeritario de Leon found that excessive fasting was associated with longer LOS adjusting for sex, age, nutritional status, infused volume, electrolytic, glucose and disease. [12] Another study investigating outcomes of nutritional parameters found that in-hospital fasting of three of more nonconsecutive days was associated with prolonged hospital stay. [13]

Our results indicate no association between gaps in care and 90-day readmission. Some evidence in the literature contradicts this finding. A program in a community hospital included discharge planning as one of its interventions and found a decrease in readmission (16.5 to 7.1%). [3] Additionally, a meta-analysis of 6 randomized clinical trials found that the odds of readmission were significantly lower for those who received oral nutritional supplements (ONS) at discharge vs routine care. A majority of the adults in our sample, with a discharge instruction gap, had the ONS omitted from their discharge instructions. [14] However, gaps were also determined for patients whose diet instructions were not restrictive enough, were overly restrictive, had inappropriate texture or had an inaccurate EN/PN order. [6] Some of these discharge issues may have more impact on health outcomes than others and a future study with larger numbers should investigate their individual association with readmission. Additionally, our findings may suggest that ensuring appropriate discharge instructions is not enough to prevent readmission and that post discharge interventions could be more effective. A nutritional intervention found that participants who received an in-home follow-up to their discharge instructions had a lower risk of 30-day and 90-day readmission compared to those who received no visit. [15]

The extant literature has shown that malnourished patients are associated with worse outcomes and higher healthcare costs. [1, 16, 17] Once identified, attention should be focused on the quality of care for malnourished patients. There is evidence to suggest the appropriate nutritional interventions are cost effective. [18–20] A systematic review found that among 12 studies comparing ONS with routine care, the mean reduction in cost for ONS was 12.2%. [21] Additionally, when properly carried out, communication and discharge interventions for the malnourished can help reduce negative health outcomes. [3] From this study, we add to the literature by pointing out that the gaps in testing and procedures also warrant consideration. Previous investigations suggest that NPO status is often prescribed to patients prematurely in anticipation of a clinical intervention that requires fasting or is ordered for a procedure in which fasting was not necessary. Hypotheses for why NPO status is inappropriately prolonged include the failure of some clinicians in adopting evidence base guidelines and a “just in case” attitude. [22] Better education of the clinical team on pre- procedure fasting guidelines may help mitigate prolonged nil per os. [13, 22–24]

Due to the unique nature of our study, there is limited ability to compare our findings with previous studies. Additionally, due to limited resources, our study had a small sample size and therefore lack of association between readmission and gaps in care may be due to type 2 error. As the study was a retrospective chart review, barriers also include limited availability of data for collection, difficulties interpreting documentation and difficulty establishing cause and effect. Our study was performed in one medical center, which could restrict generalizability. Further research should be conducted at other institutions. While the feasibility of defining malnutrition through the A.S.P.E.N./AND criteria has been assessed, it has yet to be validated in a large sample. [25, 26] However, complete data collection for all participants in the study ensured that there was no missing data and therefore little bias due to missing information. Two clinical dietitians reviewed and examined a selection of charts and discussed discrepancies, which added to the strength of this study.

## Conclusion

While it is of the utmost importance to identify patients with malnutrition in hospitals, it is equally important to understand what can be done to mitigate negative outcomes among such malnourished patients. Understanding gaps in care for those diagnosed with malnutrition and how these gaps relate to length in stay and readmission is vital for focusing the implementation of future policies and interventions among this vulnerable population. In order to reduce gaps in care, improved processes for scheduling tests/procedures to prevent prolonged NPO status need to be addressed. Collaboration between departments within hospitals to identify and clearly establish which specific procedures necessitate NPO status and the time period for which NPO is required is critical and could help eliminate this gap. Clear and constant communication among the primary care team and the dietitian is also necessary to overcome existing gaps in care. Additionally, we recommend that a reconciliation of nutrition related discharge orders be conducted prior to discharge.

## Abbreviations

A.S.P.E.N.: American Society for Parenteral and Enteral Nutrition
AND: Academy of Nutrition and Dietetics
CI: Confidence Interval
CMS: Centers for Medicare and Medicaid Services
EN/PN: Enteral or Parenteral
ICD9: International Classification of Diseases, Ninth Revision
IQR: Interquartile Range
LOS: Length of stay
NPO: nil per os
ONS: Oral Nutritional Supplements
OR: Odds Ratio
